# The effect of a one-time 15-minute guided meditation (Isha Kriya) on stress and mood disturbances among operating room professionals: a prospective interventional pilot study

**DOI:** 10.12688/f1000research.18446.1

**Published:** 2019-03-26

**Authors:** Valluvan Rangasamy, Ammu Thampi Susheela, Ariel Mueller, Tracy F. H. Chang, Senthilkumar Sadhasivam, Balachundhar Subramaniam

**Affiliations:** 1 Department of Anesthesia, Critical Care, and Pain Medicine, Beth Israel Deaconess Medical Center, Harvard Medical School, Boston, Massachusetts, 02215, USA; 2 Department of Labor Studies and Employment Relations (LSER),, Rutgers University, New Brunswick, NJ, USA; 3 Department of Anesthesiology, Riley’s Hospital for Children, Indiana University School of Medicine,, Indianapolis,, IN, USA

**Keywords:** meditation, stress, burnout, healthcare providers, anesthesia, surgery, operating room professionals

## Abstract

**Background: **Operating room professionals are exposed to high levels of stress and burnout. Besides affecting the individual, it can compromise patient safety and quality of care as well. Meditation practice is getting recognized for its ability to improve wellness among various populations, including healthcare providers.

**Methods: **Baseline stress levels of perioperative healthcare providers were measured via an online survey using a Perceived Stress Scale (PSS) questionnaire. An in-person meditation workshop was demonstrated during surgical grand rounds and an international anesthesia conference using a 15-minute guided Isha Kriya meditation. The participants were then surveyed for mood changes before and after meditation using a Profile of Mood States (POMS) questionnaire.

**Results: **Surgeons and anesthesiologists were found to have higher median (interquartile range) Perceived Stress Scores as compared to nurses respectively (17 [12, 20] and 17 [12, 21] vs 14 [9, 19]; *P *= 0.01). Total mood disturbances were found to be significantly reduced after meditation in both the surgical grand rounds (pre-meditation median [IQR] 99 [85, 112] vs 87 [80, 93] post-meditation; *P* < 0.0001) and anesthesia conference cohorts (pre-meditation 92 [86, 106] vs 87 [81, 92] post-meditation; *P* < 0.0001).

**Conclusions: **Isha Kriya, a guided meditation, is easy to learn and takes less than 15 minutes to complete. This meditation technique improves mood changes and negative emotions among operating room professionals and could be used as a potential tool for improving wellness.

## Introduction

Wellness is not just merely an absence of a disease, it encompasses physical, mental and social well-being ^1^. Stress associated with work, when left unaddressed, results in burnout. Around 30–60% of healthcare providers suffer from burnout and 400 physicians die by suicide every year ^2^. Work environment (work overload, insufficient reward), demographic variables (early career, lack of life partner or children), and personality traits (low confidence, overachieving, impatience, perfectionist) can contribute to stress and burnout ^2^.

Operating rooms are highly stressful environments involving complex, respectful, and essential interactions. Surgeons, anesthesiologists and nurses can develop health problems, social issues and substance use due to stress and burnout ^3, 4^. Employee disengagement is estimated to cost around 450 billion USD per year, and illness-related absences are common in burned out employees ^5^. Despite the critical need, no effective treatment options and well-described organizational approach exists for wellness among healthcare providers ^6^.

World Health Organization (WHO) emphasizes the need for workplace health interventions aiming to improve the wellness of employees ^1^. However, there is a lack of high-quality trials with simple and potentially effective interventions. Isha Kriya (IK) is a 15-minute, simple guided meditation tool employing thought, breathing, and awareness ^5^. We hypothesized that operating room professionals experience significant stress, and IK meditation would decrease their mood disturbances. In this pilot study, we sought to measure the a) stress levels among surgeons, nurses and anesthesiologists, and b) mood changes following a one-time IK meditation session.

## Methods

### Ethical considerations and reporting

This prospective interventional pilot study was conducted after Institutional Review Board approval (Beth Israel Deaconess Medical Center, Boston, MA, US. Protocols 2017P000657, 2018P000200, 2018P000585) with an electronic consent. Written informed consent was waived by our IRB. This manuscript adheres to the applicable Consolidated Standards of Reporting Trials (CONSORT) statement for pilot and feasibility trials ^7, 8^.

### Stress assessment

An online survey with Perceived Stress Scale (PSS) questionnaire (available as extended data ^9^) was used to assess the stress levels among the operating room professionals at a single academic teaching hospital. This 10-item questionnaire measures an individual’s feeling about a stress factor over the past 30 days ^10, 11^. Anesthesiologists were surveyed in February 2018, and other operating room professionals including nurses and surgeons were surveyed in August 2018. The survey was conducted anonymously and willing participants could complete using Research Electronic Data Capture (REDCap 8.10.6; Vanderbilt University) with three weekly automatic reminders for incomplete surveys.

### Meditation and evaluation

Following this survey, a 15-minute guided IK meditation (guide available as extended data ^12^) was demonstrated during a) surgical grand rounds at our institution (September 2018) and b) the Annual Meeting of American Society of Anesthesiologists (ASA) wellness workshop (October 2018). The Profile of Mood States (POMS) [Revised Version 15.2] ^13^ questionnaire (available as extended data ^14^) was used to assess the mood changes before and after meditation. POMS is a psychological rating scale assessing transient and distinct mood states. In both the settings, willing participants could complete the survey anonymously either on paper or by using a link directly in REDCap.

### Statistical analysis

Descriptive data were presented as median (interquartile range) or frequencies and proportions. Normality was assessed using Shapiro-Wilk test. Differences between cohorts were assessed using Wilcoxon-Mann-Whitney or chi-square test, as appropriate. With small cell sizes, Fisher’s Exact test was used. No sample size calculations were done in this pilot trial. All willing members in the specific settings were included in the study.

Our primary outcome, the mood changes with meditation, was assessed using separate Wilcoxon signed rank sum tests. Bonferroni correction was used to keep the familywise error rate at 0.05. *P* values were considered statistically significant when < 0.025 (0.05 / 2 tests). Analysis of secondary outcomes including positive and negative subscales with their respective components were deemed exploratory and *P* values < 0.05 were considered statistically significant. All data were analyzed using SAS 9.4 (SAS Institute Inc., Cary, NC).

## Results

### PSS Survey

Table 1 displays the demographic information from the PSS survey. Of the total 910 participants, 362 (39.8%) completed the survey, including 101 (27.9%) anesthesiologists, 61 surgeons (16.9%) and 151 (41.7%) nurses. Respondents were primarily female (65.8%), white (73.1%), non-Hispanic/Latino (87.6%) and 30–44 years old (38%). One-third (32.8%) of respondents had more than 15 years of work experience. Nurses reported more years of work experience than the other respondents (47.7% vs 23% and 22.2%; *P* < 0.0001). Raw data from this survey are available on Figshare ^15^.

**Table 1.** Demographics of Perceived Stress Scale (PSS) Survey Respondents.

Demographics	Surgeons *N = 61*	Anesthesiologists *N = 101*	Nurses *N = 151*	*P*-Value
**Age,** *years*				0.22
18–29	11 (18.33)	15 (15.15)	21 (13.91)	
30–44	27 (45.00)	47 (47.47)	48 (31.79)	
45–54	9 (15.00)	21 (21.21)	33 (21.85)	
55–64	9 (15.00)	12 (12.12)	38 (25.17)	
65 & older	3 (5.00)	3 (3.03)	9 (5.96)	
I prefer not to answer	1 (1.67)	1 (1.01)	2 (1.32)	
**Female Gender**	23 (37.70)	46 (46.94)	136 (90.07)	<0.0001
**Race**				0.004
White	41 (68.33)	66 (67.35)	130 (86.09)	
Black or African American	0 (0)	0 (0)	1 (0.66)	
Asian	12 (20.00)	15 (15.31)	7 (4.64)	
Multi-Racial	2 (2.04)	2 (2.04)	2 (1.32)	
Other	7 (7.14)	7 (7.14)	2 (1.32)	
I prefer not to answer	8 (8.16)	8 (8.16)	9 (5.96)	
**Ethnicity**				0.03
Not Hispanic or Latino	53 (91.38)	79 (83.16)	134 (91.16)	
Hispanic or Latino	3 (5.17)	4 (4.21)	0 (0)	
I prefer not to answer	2 (3.45)	12 (12.63)	13 (8.84)	
**Clinical Role**				0.98
Attending	32 (56.14)	46 (49.46)	---	
Fellow	3 (5.26)	5 (5.38)	---	
Resident	22 (38.60)	28 (30.11)	---	
Nurse	0 (0)	10 (10.75)	---	
Researcher	0 (0)	1 (1.08)	---	
I prefer not to answer	0 (0)	3 (3.23)	---	
**Years of Service**				<0.0001
0–1	6 (9.84)	18 (18.18)	7 (4.64)	
2–5	19 (31.15)	33 (33.33)	25 (16.56)	
5–10	14 (22.95)	11 (11.11)	17 (11.26)	
10–15	8 (13.11)	11 (11.11)	28 (18.54)	
> 15	14 (22.95)	22 (22.22)	72 (47.68)	
I prefer not to answer	0 (0)	4 (4.04)	2 (1.32)	
**Perceived Stress Score**	17.0 (12.0, 20.0)	17.0 (11.5, 21.0)	13.5 (9.0, 19.0)	0.01

*Values are presented as number (%) or median (quartile 1, quartile 3).*

Surgeons and anesthesiologists had higher median [IQR] perceived stress scores than nurses (17 [12, 20] and 17 [11.5, 21] vs 13.5 [9, 19]; *P* = 0.01). Table 2 compares the perceived stress scores of based on the clinical role. Surgery residents (17 [15, 19]) and fellows (18.5 [14, 20]) had higher stress scores than attending physicians (17.5 [10, 20]), although this difference was not significant ( *P* = 0.12). A similar relationship was observed among residents and fellows in anesthesia ( *P* = 0.71). **

**Table 2.** Perceived Stress Scores Among Surgeons and Anesthesiologists Stratified by Clinical Role.

Clinical Role	Attending	Resident	Fellow	*P*-Value
Surgeons	17.5 (10.0, 20.0)	17.0 (15.0, 19.0)	18.5 (14.0, 20.0)	0.12
Anesthesiologists	16.0 (11.0, 18.0)	20.0 (14.0, 24.0)	20.0 (13.0, 24.0)	0.71

*Values are presented as median (quartile 1, quartile 3).

### POMS survey

Demographic data for participants who completed the POMS survey were listed in Table 3. A total of 50 surgeons participated in IK meditation during surgical grand rounds and 28 (56%) completed the POMS questionnaire. Raw data from this questionnaire are available on Fighsare ^16^. Similarly, 52 anesthesiologists attending the ASA conference participated in the meditation workshop and 44 (85%) completed the questionnaire. Respondents from the anesthesia conference were significantly older, more likely to be attending physicians and had more experience as compared to those from surgical grand rounds (all *P* values < 0.0001). A significantly higher number of anesthesia conference respondents reported meditating ( *P* = 0.03) or performing aerobic exercise ( *P* = 0.002) three to four times a week as compared to the surgical respondents at our institution. Only 7% of surgical and 27% of anesthesia conference respondents practiced meditation routinely.

**Table 3.** Demographic Data of Respondents Who Completed the Profile of Moods Survey (POMS).

Demographics	Surgical Grand Rounds *N = 28*	Anesthesia Conference *N = 44*	*P*-Value
**Age,** *years*			<0.0001
18–40	22 (78.57)	11 (25.00)	
40–60	3 (10.71)	18 (40.91)	
≥ 60	2 (7.14)	15 (34.09)	
I prefer not to answer	1 (3.57)	0 (0)	
**Female Gender**	11 (39.29)	26 (59.09)	0.10
**Role**			<0.0001
Attending	4 (14.29)	34 (77.27)	
Fellow	4 (14.29)	0 (0)	
Resident	13 (46.43)	3 (6.82)	
Other	6 (21.43)	6 (13.64)	
I prefer not to answer	1 (3.57)	1 (2.27)	
**Years of Service**			<0.0001
0–5	22 (78.57)	8 (18.18)	
5–10	0 (0)	4 (9.09)	
10–20	2 (7.14)	11 (25.00)	
≥ 20	1 (3.57)	21 (47.73)	
I prefer not to answer	3 (10.71)	0 (0)	
**Meditated 3–4 Times a Week Before**	2 (7.14)	12 (27.27)	0.03
**Aerobic Exercises 3–4 Times a Week**	8 (28.57)	29 (65.91)	0.002

*Values are presented as number (%).*

### Mood changes with IK meditation

**Figure 1.**
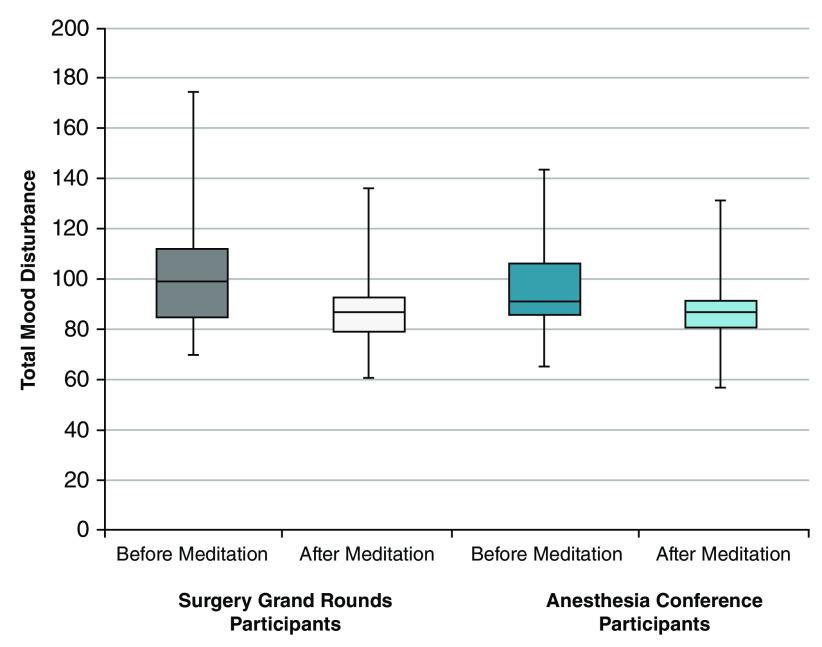
Total mood disturbances before and after Isha Kriya meditation. Each of the boxplots represents the total mood disturbance before and after a single guided meditation session (Isha Kriya) among all respondents, including those at both the surgical grand rounds and the anesthesia conference.

Mood changes before and after IK meditation during surgical grand rounds and anesthesia conference were presented in Table 4 and Figure 1. In surgical grand rounds, total mood disturbances (TMD) were significantly reduced after meditation (pre vs post meditation: median [IQR] 99 [85, 112] vs 87 [80, 93]; *P* < 0.0001). A similar reduction was observed with all negative subscales (19 [7, 32] vs 7 [2, 31]; *P* < 0.0001), including tension, anger, fatigue, confusion and depression (all statistically significant; *P*-values < 0.003). No significant change was observed for positive subscales (22 [17, 26] vs 22 [18, 27]; *P* = 0.94), including esteem-related affect and vigor.

**Table 4.** Outcomes of Respondents Who Completed the Profile of Moods Survey (POMS) Before and After Isha Kriya Meditation.

Outcomes	Surgical Grand Rounds *N = 28*			Anesthesia Conference *N = 44*		
	Before Meditation	After Meditation	*P-*value	Before Meditation	After Meditation	*P-*value
**Total Mood Disturbance**	99 (85, 112)	87 (80, 93)	<0.0001 *	92 (86, 106)	87 (81, 92)	<0.0001 *
**Individual Subscales**						
*Negative Subscales*	19 (7, 32)	7 (2, 13)	<0.0001 *	14 (11, 23)	4 (1, 9)	<0.0001 *
Tension	5 (2, 8)	1 (0, 3)	<0.0001 *	4 (1, 6)	0 (0, 3)	<0.0001 *
Anger	1 (0, 5)	0 (0, 1)	0.003 *	1.5 (0, 3)	0 (0, 0)	<0.0001 *
Fatigue	6 (2, 8)	3 (1, 5)	0.0001 *	4 (3, 7)	4 (1, 9)	<0.0001 *
Depression	2 (0, 5)	0 (0, 1)	0.0003 *	2 (0, 4)	0 (0, 0)	<0.0001 *
Confusion	4 (2, 6)	1 (0, 3)	<0.0001 *	4 (2, 6)	1 (0, 2)	<0.0001 *
*Positive Subscales*	22 (17, 26)	22 (18, 27)	0.94	22 (17, 28)	20 (15, 24)	0.15
Esteem Related Affect	15 (13, 18)	16 (12, 17)	0.99	15 (12, 17)	15 (12, 17)	0.69
Vigor	6.5 (6, 9)	7 (5, 9)	0.98	7 (4, 11)	6 (2, 8)	0.03

Values are presented as median (quartile 1, quartile 3). Note: In order to account for multiple testing of our primary outcome, total mood disturbance, p-values for the primary outcome are significant if the p-value is < 0.025 (= 0.05 / 2). All other secondary outcomes including individual subscales are considered exploratory, therefore p-values < 0.05 are considered statistically significant. Significant values are denoted as * in the table above.

Consistent with the results from surgical respondents, TMD during anesthesia conference significantly reduced after meditation (92 [86, 106] vs 87 [81, 92]; *P* < 0.0001). Negative subscales showed a significant reduction after meditation (14 [11, 23] vs 4 [1, 9]; *P* < 0.0001). Individual subscales such as tension, anger, fatigue, confusion, and depression were also reduced significantly (all *P* values < 0.0001). No significant changes were seen for positive subscales.

## Discussion

This study showed two important findings: a) high levels of stress among operating room professionals and b) one-time 15-minute guided IK meditation significantly reduced TMD, including negative subscales such as tension, anger, fatigue, depression and confusion.

Stress levels among physicians in this study were higher than those levels reported in the general population ^10^. In a 2011 national survey, physicians had higher levels of stress and burnout than any other working population in the United States ^17^. Excessive workload, administrative burdens, decreased meaning from work, and difficulty in managing personal life were some of the prominent reasons suggesting a significant workplace contribution for burnout.

The higher stress levels found among surgical residents and fellows in training than attending physicians were consistent with a nationwide survey from France that showed a 40% incidence of severe burnout in surgery residents ^18^. Those authors insisted on further research and interventions to prevent any devastating effects among residents. In this study, we explored the effectiveness of guided IK meditation and found a significant improvement in mood changes.

Mindfulness and meditation techniques were utilized for the wellness of healthcare providers ^19^. The estimated cost of burnout among physicians in Canada was found as $213 million ^20^. Harvard business review reports that Johnson & Johnson saved around $250 million on health care costs by implementing in-house wellness programs ^21^. In this study, after a 15-minute, simple, guided IK meditation, participants reported a reduction in mood disturbances. It is encouraging to compare these results with previous research where healthcare professionals reported widespread benefits after meditation programs ^19^. However, the participants in those studies were from various parts of healthcare system, where the levels of stress and burnout could be different.

The operating room environment is unique, and conflicts may arise due to a difference in information, opinion, experience and interests. IK meditation could be used as a simple tool to de-identify thoughts and bodily sensations.

Our study has several limitations. Although this study utilized anonymous surveys, participation bias becomes unavoidable. The participation was kept entirely voluntary, and self-selection bias could limit extrapolation of the observed results. Furthermore, our sample size was relatively small, which may have implications in the generalization of these results. To our knowledge, this study was the first to use a simple, one-time, 15-minute guided IK meditation for improving wellness among operating room professionals. We used a validated PSS and POMS questionnaire to observe the perceived stress level as well as mood changes after meditation.

In conclusion, our findings suggest that IK, a 15-minute guided meditation technique, could improve mood changes among operating room professionals. Stress levels observed among physicians emphasize the need for strategies aiming to improve wellness. Moreover, randomized trials and research observing long term effect of meditation practice compliance and positive provider wellbeing that will eventually translate to better patient outcomes.

## Data availability

### Underlying data

Figshare: Perceived Stress Scale (PSS) Survey.xlsx. https://doi.org/10.6084/m9.figshare.7808723 ^15^.

Figshare: Profile of Mood States (POMS) survey before and after Isha Kriya meditation.xlsx. https://doi.org/10.6084/m9.figshare.7808729 ^16^.

### Extended data

Figshare: Perceived Stress Scale (PSS) survey questionnaire. https://doi.org/10.6084/m9.figshare.7808747 ^9^.

Figshare: Instructions and details of IshaKriya meditation session. https://doi.org/10.6084/m9.figshare.7808741 ^12^.

Figshare: Profile of Mood States (POMS) questionnaire https://doi.org/10.6084/m9.figshare.7808756 ^14^.

### Reporting guidelines

Figshare: CONSORT checklist, extension for Pilot and Feasibility Trials for study “The effect of a one-time 15-minute guided meditation (Isha Kriya) on stress and mood disturbances among operating room professionals: a prospective interventional pilot study”. https://doi.org/10.6084/m9.figshare.7808735 ^8^.

Data are available under the terms of the Creative Commons Attribution 4.0 International license (CC-BY 4.0).
