# Supplementary material for: The effect of a one-time 15-minute guided meditation (Isha Kriya) on stress and mood disturbances among operating room professionals: a prospective interventional pilot study
Source: F1000Res. 2019 Mar 26;8:335. [Version 1] doi: 10.12688/f1000research.18446.1 (PMC7327726; doi:10.12688/f1000research.18446.1)
Supplement: Peer review history

# Peer review: Reviewer response for version 1

**DOI:** 10.5256/f1000research.20181.r64639

**Published:** 2020-06-30

**Authors:** Salvatore Zaffina

**Affiliations:** 1 Occupational Medicine Unit, Bambino Gesù Children’s Hospital - IRCCS, Rome, Italy

The topic of the article is the use of meditation as intervention for the health and wellness promotion of healthcare professionals of operating room. Stress levels observed among physicians emphasize the need for strategies aiming to improve wellness.

 Meditation practice is getting recognized for its ability to improve wellness among various populations, including healthcare providers. Before the intervention the researchers provide a first measurement of stress of healthcare professionals of operating room. An in-person meditation workshop was demonstrated during surgical grand rounds and an international anesthesia conference using a 15-minute guided Isha Kriya meditation. The participants were then surveyed for mood changes before and after meditation using a Profile of Mood States questionnaire. The results of the pre-post measurements showed an improvement of mood changes and negative emotions.

 The study is interesting and well conducted. The reviewer suggests some integrations and corrections described in the following comments.

 In the abstract the Perceived Stress Scores of nurses presented a different value from that reported in the result section (14 instead of 13.5). In the abstract and all the manuscript change P<0.0001 with p<0.001 without spaces and P with p.

 In the abstract sections and methods briefly describe the hospital where the study was conducted.

 In the second line of results, the total number of participants in the three categories is 313 and not 362 (101 anesthesiologists, 61 surgeons and 151 nurses). Re-calculate the percentage by category and the resulting values of the other variables.

 In the discussion on page 7 at the beginning of the first column, the authors should briefly compare their findings with some recently published studies on the use of mindfulness in healthcare professionals.

 Pérez-Fuentes MDC, Gázquez Linares JJ, Molero Jurado MDM *et al. *The mediating role of cognitive and affective empathy in the relationship of mindfulness with engagement in nursing (2020) ^1^

 La Torre G, Raffone A, Peruzzo M *et al. *Yoga and Mindfulness as a Tool for Influencing Affectivity, Anxiety, Mental Health, and Stress among Healthcare Workers: Results of a Single-Arm Clinical Trial (2020) ^2^

 Ducar DM, Penberthy JK, Schorling JB *et al. *Mindfulness for healthcare providers fosters professional quality of life and mindful attention among emergency medical technicians (2020) ^3^

 Klein A, Taieb O, Xavier S, Baubet T, Reyre A. The benefits of mindfulness-based interventions on burnout among health professionals: A systematic review. (2020) ^4^

Is the work clearly and accurately presented and does it cite the current literature?

Partly

If applicable, is the statistical analysis and its interpretation appropriate?

Yes

Are all the source data underlying the results available to ensure full reproducibility?

Yes

Is the study design appropriate and is the work technically sound?

Yes

Are the conclusions drawn adequately supported by the results?

Yes

Are sufficient details of methods and analysis provided to allow replication by others?

Yes

Reviewer Expertise:

Occupational medicine, Workplace health promotion, Public Health, Work related stress

I confirm that I have read this submission and believe that I have an appropriate level of expertise to confirm that it is of an acceptable scientific standard.

## References

[1] : The mediating role of cognitive and affective empathy in the relationship of mindfulness with engagement in nursing. *BMC Public Health*.2020;20(1) : 10.1186/s12889-019-8129-7 16 10.1186/s12889-019-8129-7 31910847PMC6947930

[2] : Yoga and Mindfulness as a Tool for Influencing Affectivity, Anxiety, Mental Health, and Stress among Healthcare Workers: Results of a Single-Arm Clinical Trial. *J Clin Med*.2020;9(4) : 10.3390/jcm9041037 10.3390/jcm9041037 32272758PMC7230658

[3] : Mindfulness for healthcare providers fosters professional quality of life and mindful attention among emergency medical technicians. *Explore (NY)*.16(1) : 10.1016/j.explore.2019.07.015 61-68 10.1016/j.explore.2019.07.015 31471216

[4] : The benefits of mindfulness-based interventions on burnout among health professionals: A systematic review. *Explore (NY)*.16(1) : 10.1016/j.explore.2019.09.002 35-43 10.1016/j.explore.2019.09.002 31727578

# Peer review: Reviewer response for version 1

**DOI:** 10.5256/f1000research.20181.r63520

**Published:** 2020-05-26

**Authors:** Akshay Anand

**Affiliations:** 1 Neuroscience Research Lab, Department of Neurology, Postgraduate Institute of Medical Education and Research, Chandigarh, Haryana, Punjab, India

The manuscript entitled, ‘The effect of a one-time 15-minute guided meditation (Isha Kriya) on stress and mood disturbances among operating room professionals: a prospective interventional pilot study’ reports an interesting finding and is well written. Following comments will further help in improving the manuscript.

 Comments

1. Out of the 362 participants who completed the PSS, the data is presented for 313 participants including 101 anesthesiologists, 61 surgeons and 151 nurses. Who were the remaining 49 participants? Why were they excluded from the study?
2. In Table 1, the total number of participants in each column i.e. surgeons, anaesthesiologists and nurses under the categories/parameters such as age, race, ethnicity, clinical role and years of service does not match with number of participant i.e. 101 anesthesiologists, 61 surgeons and 151 nurses included in the study. For example, the sum of participants under surgeon column and race parameter is 70, which is more than the 61 surgeons who had completed the PSS.

Is the work clearly and accurately presented and does it cite the current literature?

Yes

If applicable, is the statistical analysis and its interpretation appropriate?

Yes

Are all the source data underlying the results available to ensure full reproducibility?

Yes

Is the study design appropriate and is the work technically sound?

No

Are the conclusions drawn adequately supported by the results?

Yes

Are sufficient details of methods and analysis provided to allow replication by others?

Yes

Reviewer Expertise:

Yoga, Neurodegeneration, Amyotrophic Lateral Sclerosis, Retinal injury, Age-related macular degeneration

I confirm that I have read this submission and believe that I have an appropriate level of expertise to confirm that it is of an acceptable scientific standard.
